# Impact of esketamine-based opioid-free anesthesia on quality of recovery after laparoscopic hysterectomy: a randomized controlled trial using the QoR-40 questionnaire

**DOI:** 10.1186/s12871-026-03863-3

**Published:** 2026-04-29

**Authors:** Yufeng Chen, Peng Qin, Enqi Tian, Meiling Hu, Xixi Qin, Guoping Wang

**Affiliations:** 1 Department of Anesthesiology, Changzhi People’s Hospital, Changzhi, Shanxi China; 2 Department of Anesthesiology, Changzhi Traditional Chinese Medicine Hospital, Changzhi, Shanxi China

**Keywords:** Esketamine, Opioid-free anesthesia, Recovery quality, Laparoscopic hysterectomy

## Abstract

**Objective:**

In patients undergoing laparoscopic hysterectomy, this study compared two anesthetic approaches—esketamine-based opioid-free anesthesia and opioid-based anesthesia—to assess their impact on postoperative recovery quality.

**Patients and methods:**

This prospective trial randomly assigned 91 patients undergoing laparoscopic hysterectomy to receive either esketamine-based anesthesia (induction and maintenance with esketamine, without perioperative opioids) or conventional opioid-based anesthesia. The primary endpoint was defined as the Quality of Recovery-40(QoR-40) score measured on postoperative day (POD) 1. Secondary measures comprised the QoR-40 score on POD 3, pain scores after surgery, hemodynamic variables, and adverse events.

**Results:**

On POD1, compared to the opioid group, the esketamine group exhibited a significantly elevated QoR-40 score (*p* < 0.001), but scores were similar by POD3 (*p* = 0.333). The esketamine group experienced a lower rate of postoperative nausea and vomiting (PONV) (17.8% vs. 37.0%, *p* = 0.040), whereas the two groups had comparable pain scores at every time point measured (all *p* > 0.05).

**Conclusion:**

Among laparoscopic hysterectomy patients, esketamine-based opioid-free anesthesia was linked to higher POD1 QoR-40 scores and fewer PONV events than opioid-based anesthesia. However, given the modest sample size, these preliminary findings warrant confirmation in larger multicenter trials.

**Trial registration:**

We prospectively registered the study protocol in the Chinese Clinical Trial Registry. (ChiCTR2300075661) on September 12, 2023.

## Introduction

Surgery and anesthesia, even when uncomplicated, can temporarily impair patients’ quality of life and comfort [1]. Poor postoperative recovery is associated with increased healthcare costs and reduced patient satisfaction [2]. Consequently, contemporary anesthetic practice increasingly prioritizes strategies that optimize recovery quality, minimize adverse events, and facilitate early return to normal function.

Intraoperative analgesia continues to rely primarily on opioids, owing to their potent analgesic efficacy. Nevertheless, these agents carry well-recognized side effects, including respiratory depression, PONV, and gastrointestinal dysmotility—all of which may compromise postoperative recovery [3]. To assess postoperative recovery, the validated QoR-40 questionnaire is available, measuring five domains: physical comfort, emotional state, physical independence, psychological support, and pain.

Its reliability, validity, and responsiveness have made it a widely adopted tool for assessing recovery outcomes following anesthesia and various surgical procedures [4].

Multimodal analgesia represents an essential part of ERAS protocols, aims to reduce opioid consumption by combining agents that target distinct nociceptive pathways. Opioid-free anesthesia (OFA) represents the most stringent implementation of this principle, defined as the complete avoidance of opioid administration—whether systemic, neuraxial, or locoregional—throughout the entire perioperative period, relying instead on non-opioid analgesics for intraoperative pain management [5, 6]. A diverse array of non-opioid agents is available for this purpose, ranging from acetaminophen and nonsteroidal anti-inflammatory drugs to adjuvants that modulate central sensitization, such as gabapentinoids and N-methyl-D-aspartate (NMDA) receptor antagonists, including esketamine [7, 8]. Opioid-sparing strategies of this nature have been associated with lower PONV incidence and faster return of bowel function, thereby facilitating adherence to ERAS pathways [9, 10].

Esketamine, the S-enantiomer of ketamine, demonstrates greater NMDA receptor affinity compared to its racemic counterpart, translating into enhanced anesthetic and analgesic potency [11]. As an emerging agent in opioid-reduced or opioid-free anesthesia, esketamine is hypothesized to reduce postoperative complications and improve recovery quality. Nevertheless, evidence specifically evaluating its impact on recovery outcomes within a completely opioid-free anesthetic regimen remains limited. The primary objective of this study was to investigate the impact of esketamine-based opioid-free anesthesia on recovery quality following laparoscopic hysterectomy, with assessment performed using the QoR-40 questionnaire.

## Methods

### Study design

Ethics approval for this double-blind, prospective randomized trial was provided by the Changzhi People’s Hospital Ethics Committee (Approval No. 202206). All enrolled participants provided written informed consent subsequent to a complete explanation of the study protocol.The trial’s design and reporting adhered to the CONSORT 2025 guidelines [12].

Eligibility criteria included patients aged 18 to 65 years, ASA physical status I–III, scheduled for laparoscopic hysterectomy at Changzhi People’s Hospital between October 2023 and April 2024. Patients were excluded if they refused participation, could not provide consent, had a history of substance abuse or psychiatric disorders, underwent emergency surgery or conversion to laparotomy, received medications outside the protocol, or experienced serious anesthetic complications (e.g., allergic reactions, malignant hypertension and bleeding) during the procedure or recovery.

A statistician not involved in patient care generated a computer-based randomization sequence (1:1 allocation; block size of 4). An independent assistant prepared sequentially numbered, tamper-evident opaque envelopes and took no part in recruitment, allocation, or outcome assessment. The attending anesthesiologist (uninvolved in sequence generation or postoperative assessment) opened the envelope immediately before anesthesia induction, ensuring allocation concealment until the latest possible moment. Intraoperative anesthesiologists were not blinded due to the intervention nature, but patients, surgeons, postoperative care staff, and outcome assessors remained blinded throughout. Patients were asked not to disclose intraoperative details to assessors.

### Anesthesia management

Upon operating room arrival, patients were connected to standard monitors: pulse oximetry, electrocardiography, noninvasive blood pressure, and bispectral index (BIS). Before induction, all patients were given intravenous midazolam 0.03 mg/kg. During surgery, the BIS was kept between 40 and 60 to maintain adequate anesthetic depth. A standardized anesthetic protocol was used for both groups. Rocuronium 0.9 mg/kg facilitated tracheal intubation. Propofol 1–2 mg/kg was given for induction, followed by continuous infusion at 4–9 mg/kg/h for maintenance. Propofol infusion rates were adjusted to keep heart rate and mean arterial pressure within 20% of baseline.

In this study, opioid-free anesthesia (OFA) is strictly defined as the complete avoidance of opioids via any route (systemic, neuraxial, or locoregional) throughout the entire perioperative period, including intraoperative maintenance and postoperative analgesia. Patients in the esketamine group were neither prescribed nor administered any medications containing opioid components at any point during the study.

In the esketamine group, anesthesia was induced with esketamine 0.5 mg/kg and maintained with a continuous infusion at the same rate. No opioids were administered intraoperatively, consistent with the OFA protocol. The esketamine infusion was stopped 30 min before surgery end to allow timely emergence, while propofol was discontinued about 5 min before skin closure.

In the opioid group, sufentanil 0.3 µg/kg was used for induction, followed by remifentanil infusion at 0.1–0.2 µg/kg/min. Both remifentanil and propofol infusions were stopped approximately 5 min before surgery end.

At surgery completion, all patients received palonosetron 0.125 mg intravenously for PONV prophylaxis. Neostigmine 2 mg combined with atropine 1 mg was administered to reverse neuromuscular blockade. Extubation occurred after patients regained consciousness, breathed spontaneously, and followed commands.

Postoperative pain was managed with a standardized rescue protocol. When the Visual Analog Scale (VAS; 0–10) at rest exceeded 4, flurbiprofen axetil 50 mg was given intravenously. If pain persisted for more than 30 min, sufentanil 0.1 µg/kg was administered as second-line rescue. The total amounts of flurbiprofen axetil and sufentanil consumed within the first 24 h were documented.

If unexpected complications (e.g., allergic reactions, malignant hypertension) occurred, the study protocol was terminated for that patient, and they were excluded from analysis.

### Outcome measurements

The primary endpoint was the QoR-40 score on POD1. The validated Chinese version of the QoR-40 questionnaire was used.

Secondary endpoints included QoR-40 score on POD3, VAS scores, analgesic requirements, intraoperative SpO₂, heart rate (HR), mean arterial pressure (MAP), and adverse events during recovery. VAS scores were recorded at three time points: PACU arrival, POD1 morning, and POD3 morning. HR and MAP were measured at baseline (5 min before induction), 1 min after induction, 1 min after intubation, and 30 min after surgery start.

Adverse events documented in the PACU included PONV, hypoxemia (SpO₂ < 90%), hemodynamic fluctuations (noninvasive blood pressure deviation > 20% from baseline), intraoperative awareness (assessed by modified Brice interview), somnolence (tendency to fall asleep without stimulation), and emergence delirium (screened using Nu-DESC). Baseline demographic and clinical characteristics (age, sex, BMI, education level, ASA status) were also recorded.

### Statistical analysis

Sample size was estimated based on prior QoR-40 studies. A 10-point difference was considered clinically meaningful [13, 14]. Assuming a standard deviation of 13, two-sided α = 0.05, and 90% power, 37 patients per group were required. Accounting for a potential 20% dropout, the target enrollment was set at 45 per group.

Data were collected in Microsoft Excel and analyzed using SPSS version 24.0 (IBM Corp., Armonk, NY, USA). Categorical variables were reported as frequencies and percentages, with between-group comparisons using chi-square or Fisher’s exact tests. Normality was assessed using the Shapiro-Wilk test. Normally distributed continuous variables were expressed as mean ± SD and analyzed by independent samples t-tests. Non-normally distributed and ordinal data were reported as median (IQR) and compared with the Mann-Whitney U test. For normally distributed data, Cohen’s d with 95% CI is reported. For non-normally distributed data, effect size r = Z/√N and Hodges-Lehmann median difference (MD) with 95% CI are reported. No adjustment was made for multiple comparisons for secondary outcomes, as these analyses were exploratory; findings should be interpreted accordingly. The statistician was blinded to group assignment. Statistical significance was defined as two-tailed *p* < 0.05.

## Results

**Fig. 1:**
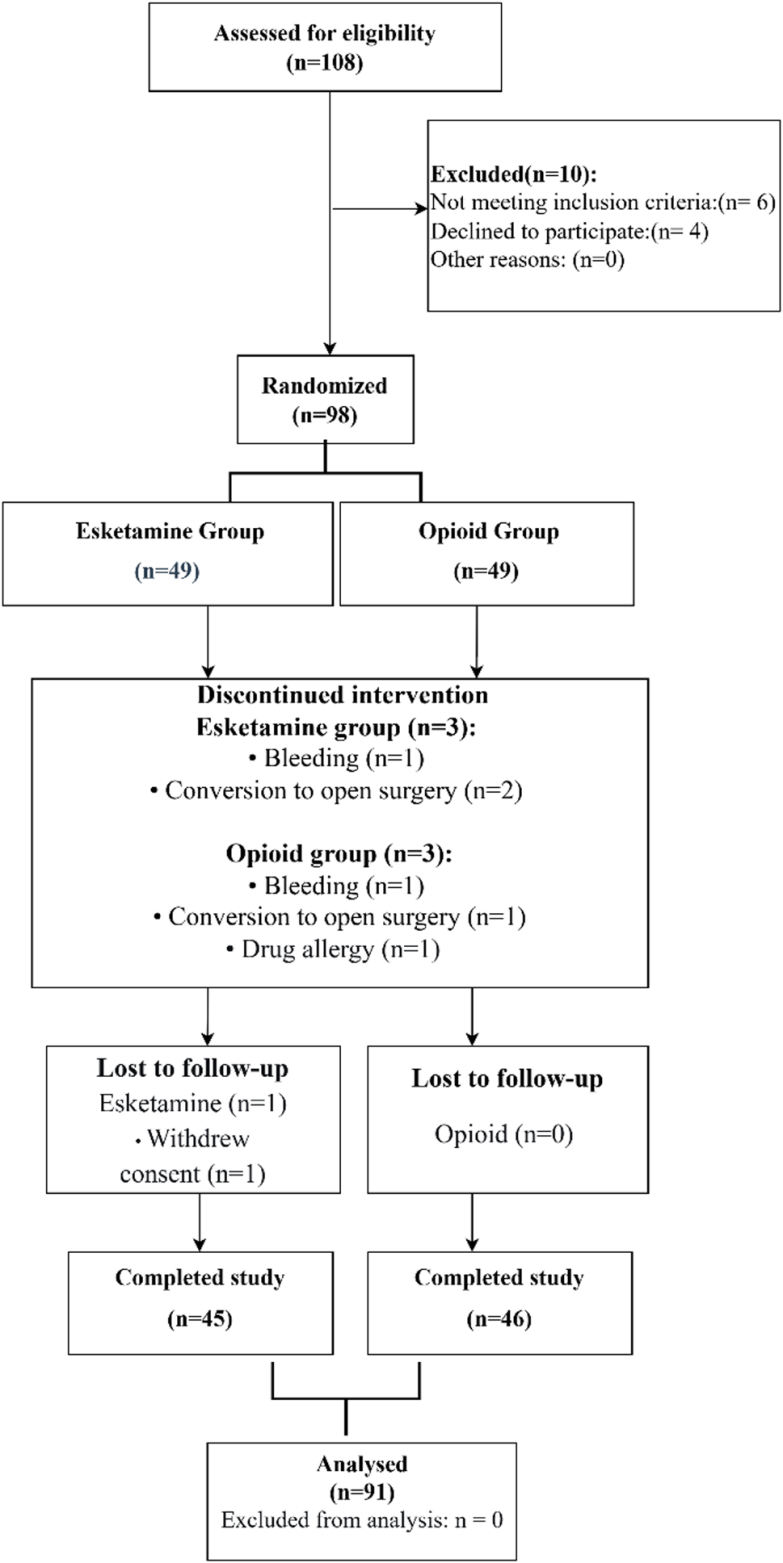
Participant flowchart

Between October 2023 and April 2024, eligibility was assessed in 108 patients. Of these, 10 were excluded, including 6 who did not meet the inclusion criteria and 4 who declined to participate. The remaining 98 patients were randomized into two groups: 49 allocated to the Esketamine group and 49 to the Opioid group. In the Esketamine group, 3 patients discontinued the intervention due to adverse events (1 case of bleeding, 2 cases of conversion to open surgery), and 1 patient was lost to follow-up (withdrew consent). As a result, 45 patients completed the study. In the Opioid group, 3 patients discontinued the intervention due to adverse events (1 case of bleeding, 1 case of drug allergy and 1 case of conversion to open surgery), and no patients were lost to follow-up. Thus, 46 patients completed the study. Overall, 91 patients were included in the final analysis. Figure 1 presents the flow diagram of patient selection. Baseline demographic and clinical characteristics were similar across the two groups (Table 1).

**Table 1:** Demographic and clinical baseline characteristics of the study population

Characteristic	Esketamine Group (*n* = 45)	Opioid Group (*n* = 46)	*P* Value
Age, years	55 (51 to 61)	54 (48 to 61)	0.303
BMI, kg/m²	24.3 (22.6 to 25.6)	24.8 (24.0 to 26.5)	0.081
ASA Physical Status			0.333
I	8 (17.8%)	6 (13.0%)	
II	36 (80%)	37 (80.4%)	
III	1 (2.2%)	3 (6.5%)	
Diabetes	10 (22.2%)	11 (23.9%)	0.848
Hypertension	15 (33.3%)	12 (26.1%)	0.449
Anemia	22 (48.9%)	24 (52.2%)	0.754
Education Level			0.621
Primary school or below	12 (26.7%)	10 (21.7%)	
Middle school	25 (55.6%)	27 (58.7%)	
College or above	8 (17.8%)	9 (19.6%)	

Data are presented as median (IQR) or n (%)

QoR-40 scores are summarized in Table 2. On POD1, the esketamine group achieved a significantly higher score than the opioid group (*p* < 0.001). By POD3, no significant between-group differences were observed in scores (*p* = 0.333).

**Table 2:** Postoperative QoR-40 and VAS Scores

Variable	Esketamine Group (*n* = 45)			Opioid Group (*n* = 46)			*P* value		Effect Size (95% CI)	
QoR-40 scores										
POD1	175 (173 to 176)			164 (163 to 167)			< 0.001^†^		MD = 11 (9 to 13) *r* = 0.86 (0.79 to 0.91)	
POD3	182 (180 to 184)			182 (179 to 183)			0.333^†^		MD = 0 (-1 to 2) *r* = 0.10 (-0.10 to 0.30)	
VAS score										
PACU	2 (2 to 3)			2 (2 to 3)			0.497^†^		MD = 0 (0 to 0) *r* = 0.07 (-0.13 to 0.27)	
POD1	2 (2 to 3)			2 (2 to 3)			0.157^†^		MD = 0 (0 to 0) *r* = 0.15 (-0.05 to 0.35)	
POD3	1 (0 to 1)			1 (0 to 1)			0.110^†^		MD = 0 (0 to 0) *r* = 0.17 (-0.03 to 0.37)	

Data are presented as median (IQR). † Mann-Whitney U test. For nonparametric comparisons, effect size r = Z/√N and median difference (MD) with 95% CI (Hodges-Lehmann) are reported

*POD* postoperative day, PACU  post-anesthesia care unit

We measured postoperative pain at rest using a 10-cm VAS at three time points: PACU arrival, POD1 morning, and POD3 morning. No significant between-group differences in VAS scores were observed at any time point (all *p* > 0.05; Table 2). Rescue analgesia was administered according to a standardized protocol (flurbiprofen axetil 50 mg for VAS > 4, with sufentanil 0.1 µg/kg as second-line if pain persisted). Rescue analgesia was required in only two patients: one in the esketamine group (PACU) and one in the opioid group (POD1). No patient required second-line rescue with sufentanil. Total flurbiprofen axetil consumption was 50 mg in each group, and no significant intergroup difference was observed in the proportion of patients requiring rescue analgesia (2.2% in both groups, *p* > 0.99, Fisher’s exact test). This result confirms that the study strictly adhered to the definition of OFA, and that patients in the esketamine group were not exposed to opioids via systemic, neuraxial, or locoregional routes throughout the entire perioperative period.

Hemodynamic parameters are presented in Table 3. Baseline values were similar between groups. Following induction, the esketamine group exhibited significantly higher MAP at all subsequent time points. HR followed a similar pattern, with no significant differences except at one minute after intubation, where values remained comparable. In summary, the esketamine group exhibited higher MAP and HR compared with the opioid group.

**Table 3:** Intraoperative hemodynamic parameters

Variable	Esketamine Group (*n* = 45)	Opioid Group (*n* = 46)		*P* value			Effect Size (95% CI)
MAP(1)	80.38 ± 5.42	82.57 ± 7.53		0.116^‡^		d = 0.33 (-0.08 to 0.74)	
MAP(2)	80.76 ± 3.98	77.00 ± 6.31		< 0.001^‡^		d = 0.77 (0.34 to 1.19)	
MAP(3)	85.47 ± 12.19	78.93 ± 7.52		0.003^‡^		d = 0.64 (0.22 to 1.06)	
MAP(4)	81.36 ± 5.58	75.93 ± 6.37		< 0.001^‡^		d = 0.91 (0.48 to 1.34)	
HR							
HR(1)	75.44 ± 8.68	75.61 ± 8.14		0.926^‡^		d = 0.02 (-0.39 to 0.43)	
HR(2)	74.00 ± 7.89	68.11 ± 6.09		< 0.001^‡^		d = 0.84 (0.41 to 1.26)	
HR(3)	82.11 ± 8.39	79.00 ± 6.54		0.051^‡^		d = 0.41 (-0.00 to 0.83)	
HR(4)	77.71 ± 6.31	66.72 ± 5.49		< 0.001^‡^		d = 1.90 (1.43 to 2.36)	

Data are presented as mean ± standard deviation. ‡ Independent samples t-test. d = Cohen’s d effect size (standardized mean difference)

MAP1 and HR1: 5 min before anesthesia induction; MAP2 and HR2: 1 min after induction; MAP3 and HR3: 1 min after intubation; MAP4 and HR4: 30 min after the start of surgery

*MAP* mean arterial pressure, HR heart rate

Adverse events during recovery are detailed in Table 4. The esketamine group had a significantly lower incidence of PONV (17.8% vs. 37.0%, *p* = 0.040). Other adverse events did not differ significantly between the two groups, including hypoxemia, hemodynamic fluctuations, somnolence, emergence delirium, or intraoperative awareness.

**Table 4:** Adverse events during emergence from anesthesia

Adverse Events	Esketamine Group (*n* = 45)	Opioid Group (*n* = 46)	*P* Value
PONV	8(17.8%)	17(37.0%)	0.040
Hypoxemia	0	0	—
Hemodynamic fluctuations	3(6.7%)	2(4.3%)	0.677
Intraoperative awareness	0	0	—
Somnolence	8(17.8%)	4(8.7%)	0.231
Emergence delirium	2(4.4%)	4(8.7%)	0.677

Data are presented as n (%). “—” indicates that the incidence was zero in both groups and statistical testing was not applicable. PONV = postoperative nausea and vomiting

## Discussion

While previous investigations of esketamine have typically combined low doses with opioids, the present study achieved complete opioid-free anesthesia (OFA) by using esketamine as the primary analgesic. Although sufentanil was permitted as a second-line rescue, it was never administered, confirming successful OFA implementation.

Several key findings emerged from this study. First, the esketamine group showed a significantly higher POD1 QoR-40 score relative to the opioid group (*p* < 0.001), but this difference was no longer apparent by POD3 (*p* = 0.333). Second, the esketamine group had a markedly lower incidence of PONV (17.8% vs. 37.0%, *p* = 0.040). Third, MAP and HR were both significantly increased in the esketamine group at multiple time points following anesthesia induction.

Our observation that esketamine improves early recovery quality is consistent with a prior meta-analysis [15]. The most direct explanations from our data are a marked reduction in PONV (17.8% vs. 37.0%)—directly enhancing the 'physical comfort' domain of the QoR-40—and more stable intraoperative hemodynamics, which could contribute to improved subjective patient well-being. Notably, despite comparable pain scores and analgesic consumption between groups, the esketamine group achieved significantly higher POD1 QoR-40 scores. This finding highlights the multidimensional nature of recovery quality captured by the QoR-40 scale (e.g., emotional state, physical independence, and nausea/vomiting), which extends beyond simple analgesia. It is known from the literature that esketamine, through NMDA receptor antagonism, provides analgesia and mitigates central sensitization [16–18]; preclinical evidence also suggests anti-inflammatory and mood-stabilizing properties [19–21]. However, we did not directly measure these parameters in the current trial, and whether these pathways contribute to the observed improvement in recovery quality remains speculative in the context of our study. The lack of sustained benefit beyond POD3 may reflect esketamine's short half-life or the specific dosing regimen used. 

Magnitude of the observed effect. The large effect size observed in our study (*r* = 0.86, 95% CI: 0.79–0.91) should be interpreted with caution, as it may be influenced by the specific clinical context and patient population. A recent BJA meta-analysis [15] reported a pooled SMD of 1.14 for esketamine on POD1 with substantial heterogeneity (I² = 69-83%), suggesting that effect sizes can vary considerably across studies. Several individual trials have reported similarly large effects. Several factors could contribute to the relatively large effect size in our study: an 11-point median difference on QoR-40 (exceeding the minimal clinically important difference), low within-group variability (IQR width 3-4 points), the comparison of opioid-free versus opioid-based anesthesia, and a relatively low baseline QoR-40 in the opioid group (median 164). However, given the single-center design and specific perioperative protocol, generalizability of this effect magnitude requires confirmation in broader clinical settings.

Patients in the esketamine group had higher heart rate and mean arterial pressure, which is consistent with the known sympathomimetic properties of esketamine. Although no adverse hemodynamic events were observed in this study, the clinical implications of these differences (e.g., the impact on patients with comorbid hypertension) remain unclear due to the limited sample size. Additionally, replacing opioids with esketamine significantly reduced PONV incidence, likely due to the opioid-sparing effect. Opioids promote PONV through both central and peripheral pathways: centrally, they stimulate the chemoreceptor trigger zone; peripherally, they bind to receptors in the gut and vestibular system, impairing gastrointestinal motility and delaying gastric emptying—a combination that potently induces vomiting [22].

Several limitations of this study warrant consideration. First, attending anesthesiologists could not be blinded due to the nature of the interventions, which may have introduced performance bias, although patients, surgeons, and outcome assessors remained blinded. Second, the discontinuation timing of anesthetic agents differed between groups (esketamine stopped 30 min before surgery end vs. remifentanil at 5 min), which may have influenced early recovery parameters, though comparable somnolence rates suggest this was successfully balanced. Third, the modest sample size (*n* = 91) limits power to detect rare adverse events and warrants cautious interpretation of the large effect size (*r* = 0.86); our single-center design in a Chinese population may also limit generalizability. Fourth, pain outcomes were subject to a floor effect (median VAS ≤ 3, rescue rate 2.2%), reflecting the mild-to-moderate pain of laparoscopic hysterectomy and limiting detection of analgesic differences. Fifth, while the QoR-40 is a validated patient-reported outcome measure, the absence of objective biomarkers (e.g., inflammatory cytokines) limits mechanistic understanding.

Despite these limitations, this randomized controlled trial offers preliminary evidence that esketamine-based OFA may improve early recovery quality in laparoscopic hysterectomy patients. These findings warrant cautious interpretation. Larger multicenter studies incorporating objective mechanistic endpoints are needed to confirm and extend these observations.

## Conclusion

In summary, this pilot study suggests that esketamine-based opioid-free anesthesia was associated with higher QoR-40 scores on POD1 and a lower incidence of PONV compared with opioid-based anesthesia. However, these preliminary findings warrant confirmation in larger multicenter trials, particularly regarding safety outcomes.

## Data Availability

The datasets generated and analyzed during the current study are available from the corresponding author upon reasonable request.The data supporting the findings of this study are available in the online supplementary materials of this article.
